# Supplementary material for: Dynamic transcriptomes identify biogenic amines and insect-like hormonal regulation for mediating reproduction in Schistosoma japonicum
Source: Nat Commun. 2017 Mar 13;8:14693. doi: 10.1038/ncomms14693 (PMC5355954; doi:10.1038/ncomms14693)
Supplement: Supplementary Information — Supplementary Figures and Supplementary Tables [file ncomms14693-s1.pdf]

Supplementary Figures and Tables

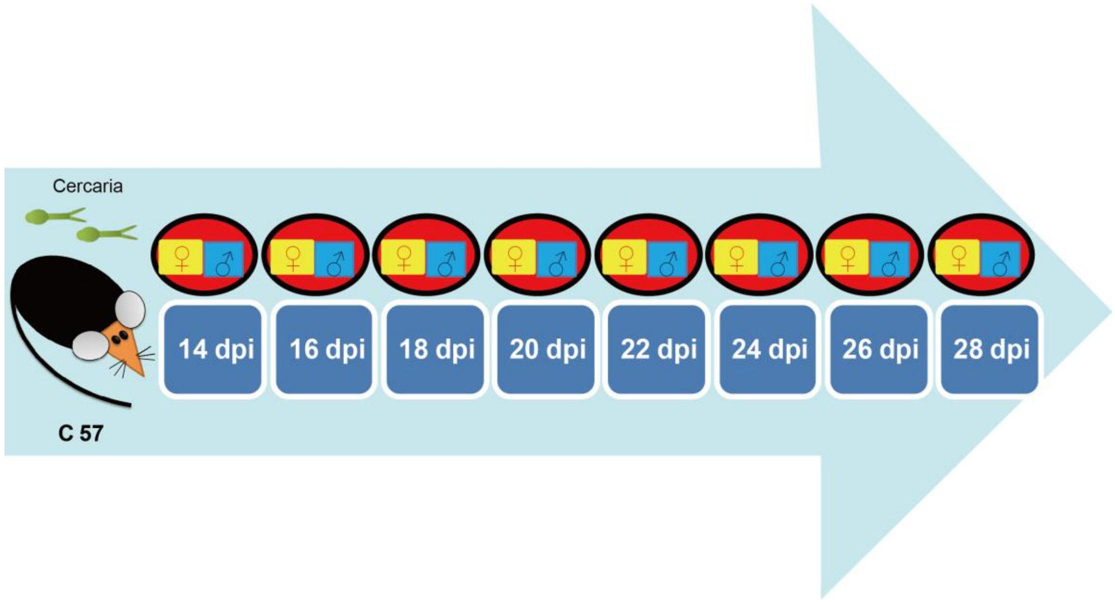

Supplementary Figure 1. Study design and sample collection. *S.japonicum* were harvested from C57 mice at 8 time points after infection. Total number of samples for RNA-Seq: 2 sexes per mouse x 3 replicates x 8 time points= 48.

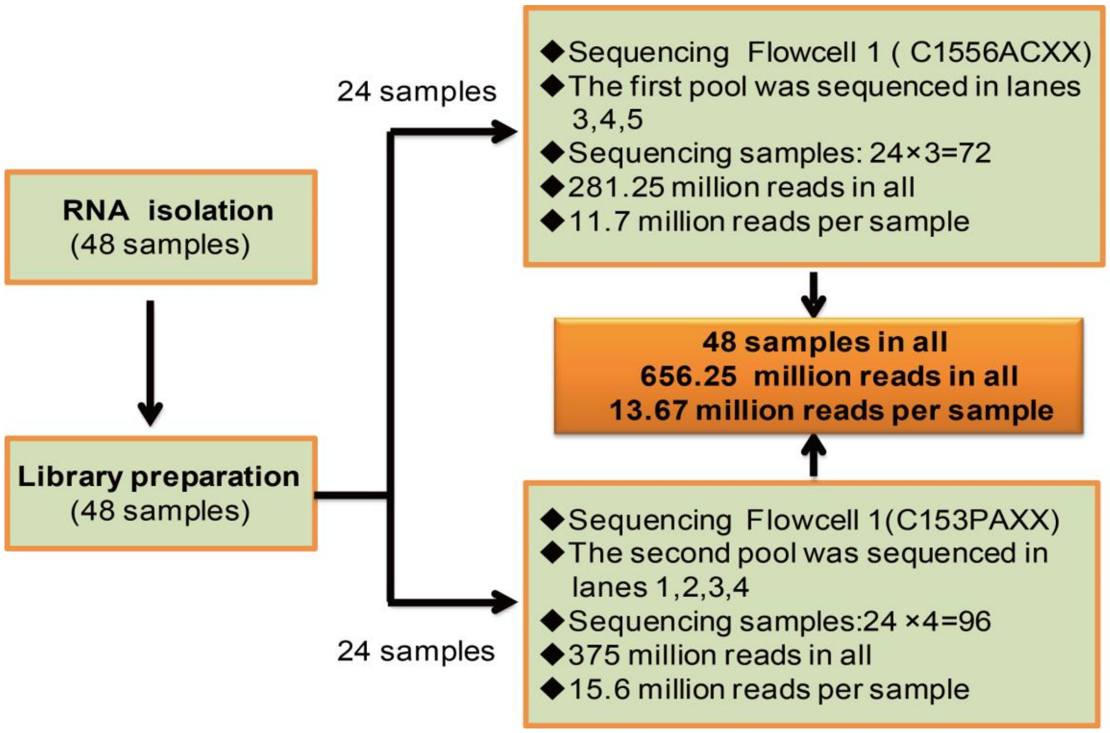

Supplementary Figure 2. High throughput RNA Sequencing. Half of 48 libraries were sequenced for 3 times, generating 11.7 million reads per sample; while the other half were sequenced for 4 times, generating 15.6 million reads per sample. In all, 48 samples generated 656.25 million reads with 13.67 million reads per sample on average.

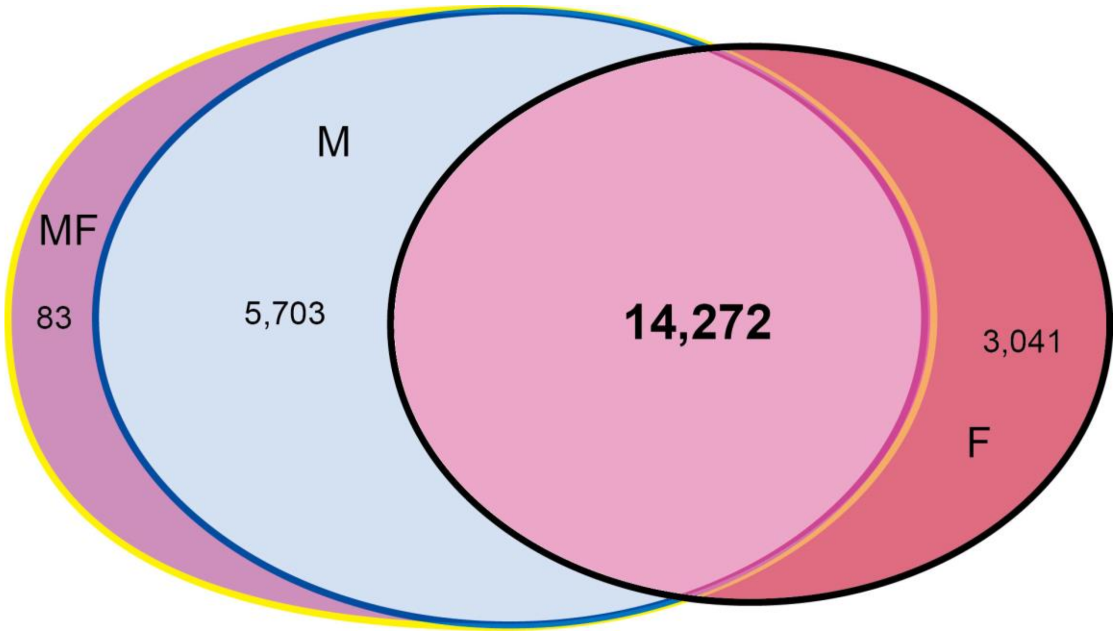

Supplementary Figure 3. Shared transcripts among three *de novo* reconstructed transcriptomes. M, transcriptome constructed from male reads. F, transcriptome constructed from female reads. MF, transcriptome constructed from male and female reads.

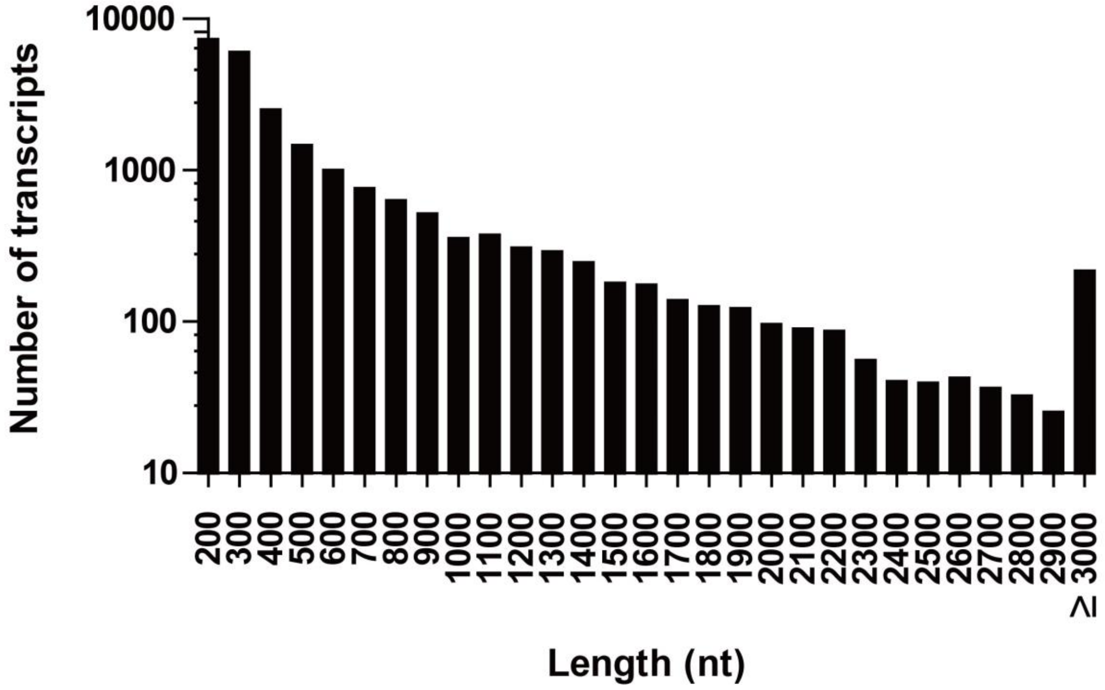

Supplementary Figure 4. Length distribution of the *de novo* reconstructed transcripts. Y-axis represents the number of transcripts in each size range and X-axis represents the size distribution.

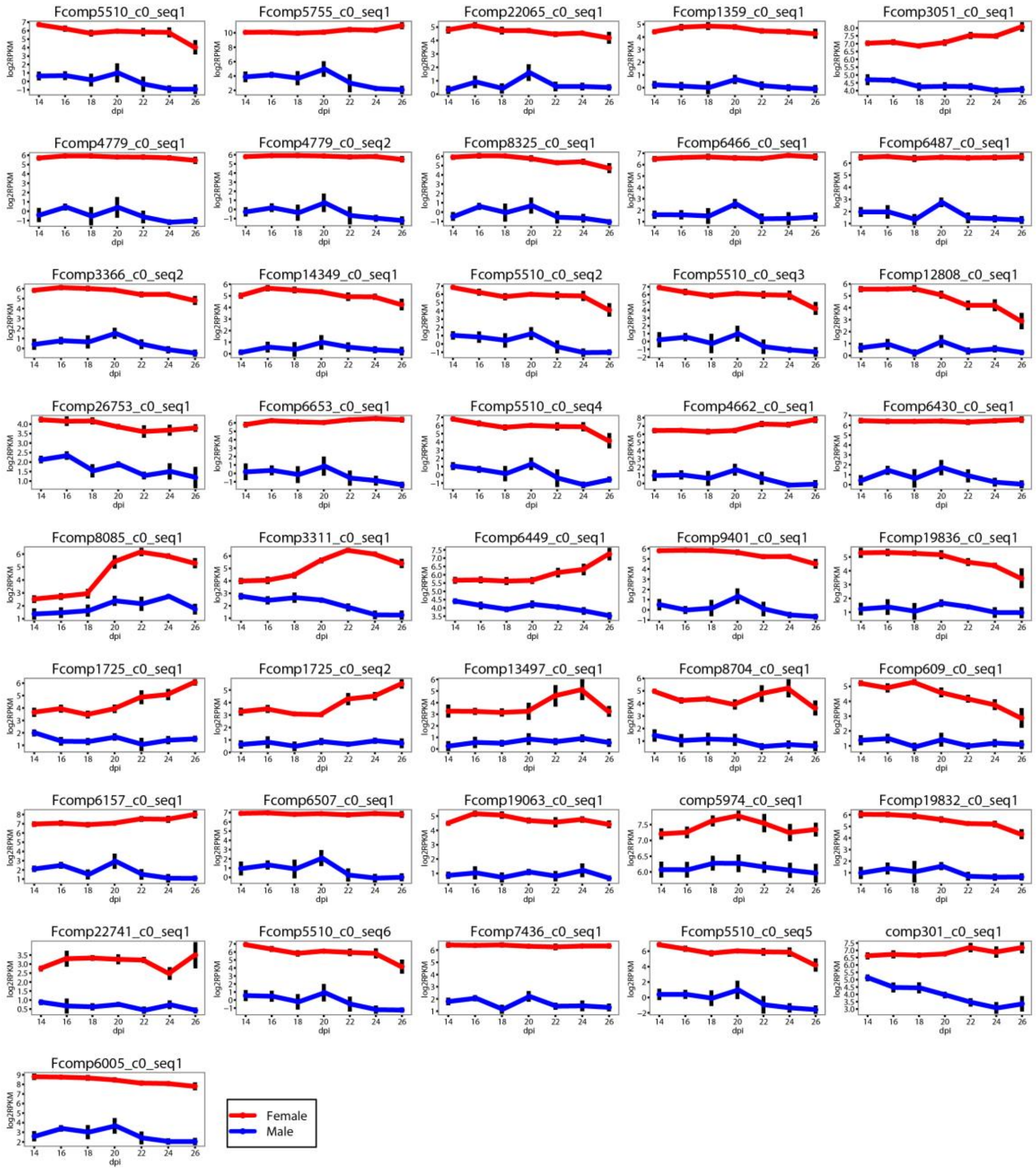

Supplementary Figure 5. The expression patterns of female-specific transcripts at 14-26 dpi. Y-axis represents the expression level (Log_2RPKM) of transcript in female (red) and male (blue). X-axis represents 7 different time points after infection. (mean±s.e.m., n=3).

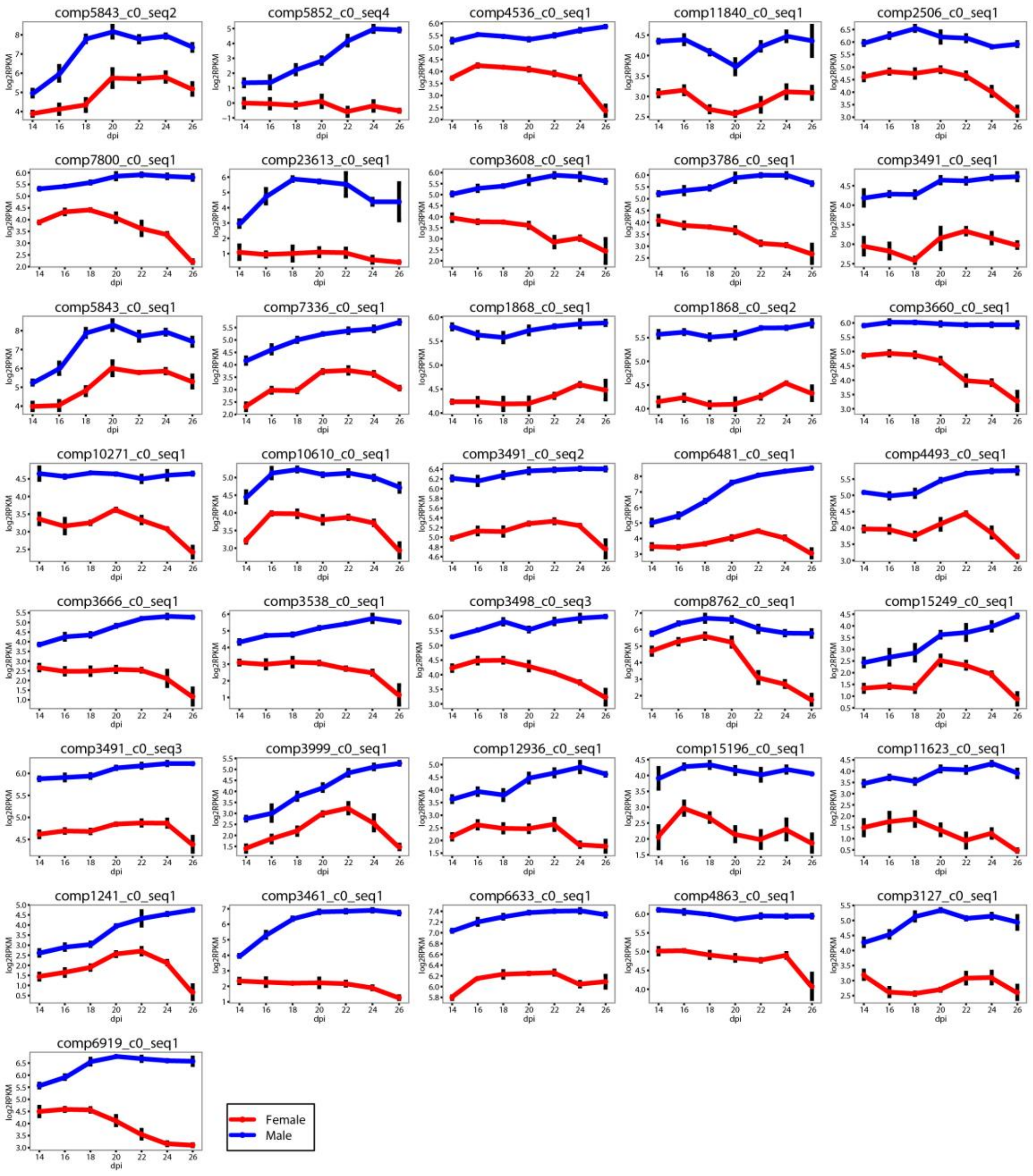

Supplementary Figure 6. The expression patterns of male-specific transcripts at 14-26 dpi. Y-axis represents the expression level (Log_2RPKM) of transcript in female (red) and male (blue). X-axis represents 7 different time points after infection. (mean ± s.e.m., n=3).

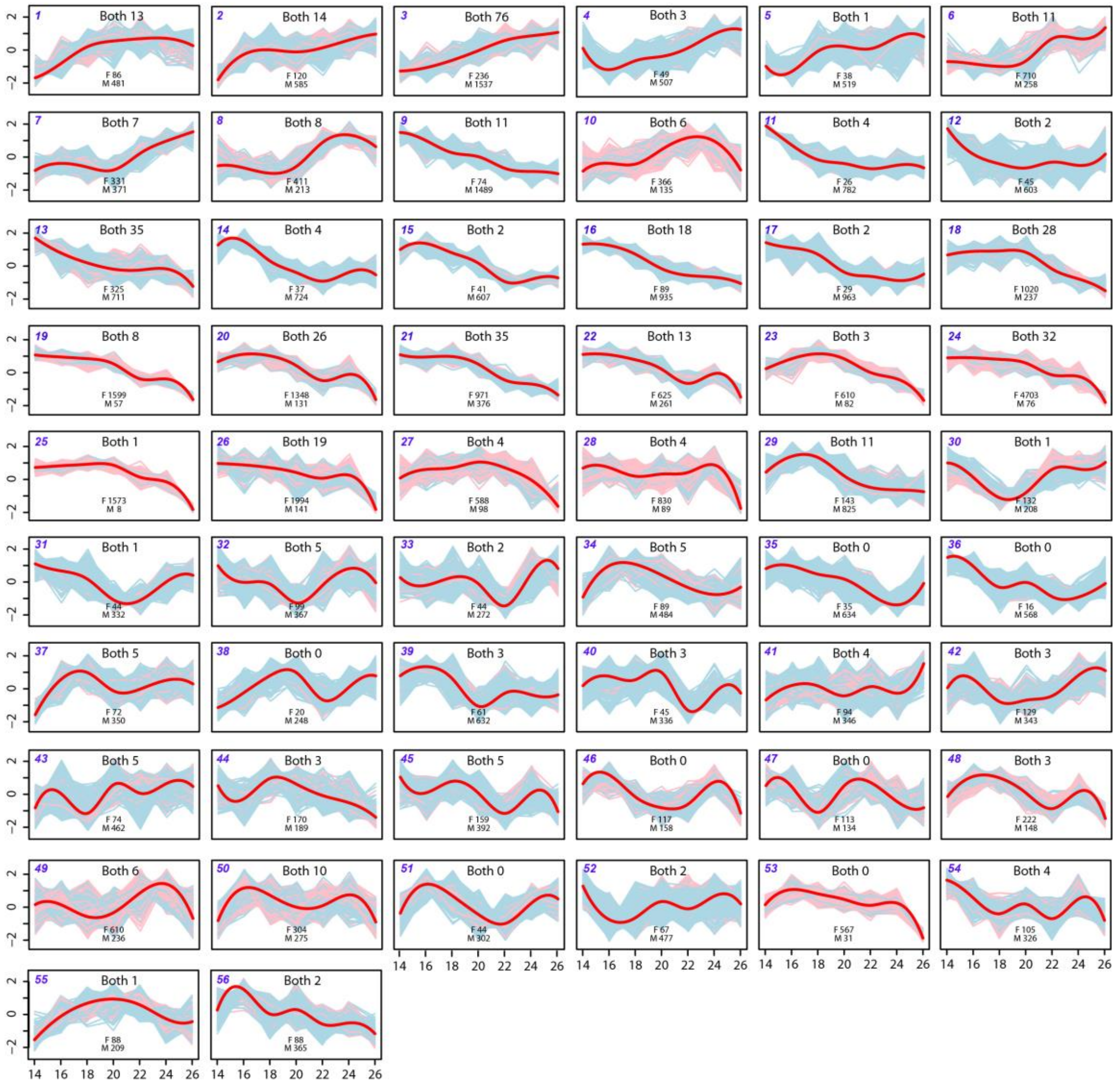

Supplementary Figure 7. Clusters of co-expressed transcripts. 56 groups in all. X-axis represents 7 different time points after infection. The thin green line (male) and thin red line (female) represents the patterns of transcripts. Bold red line represents the average intensity of clustered transcripts.

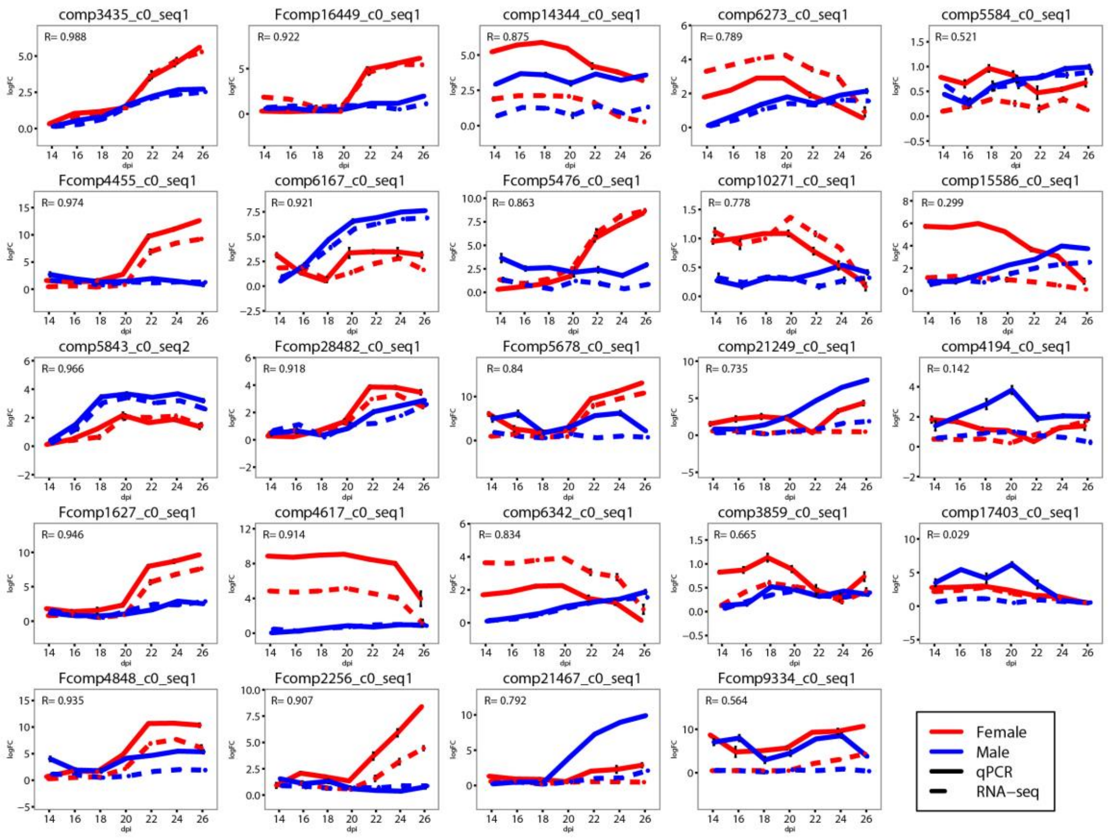

Supplementary Figure 8. Verification of RNA-Seq results by qPCR. 24 genes in all. X-axis represents 7 different time points after infection. Y-axis represents the relative expression level (Log_2 Fold change) of transcript in female (red) and male (blue) evaluated by qPCR (solid line) and RNA-seq (dotted line). R, correlation.

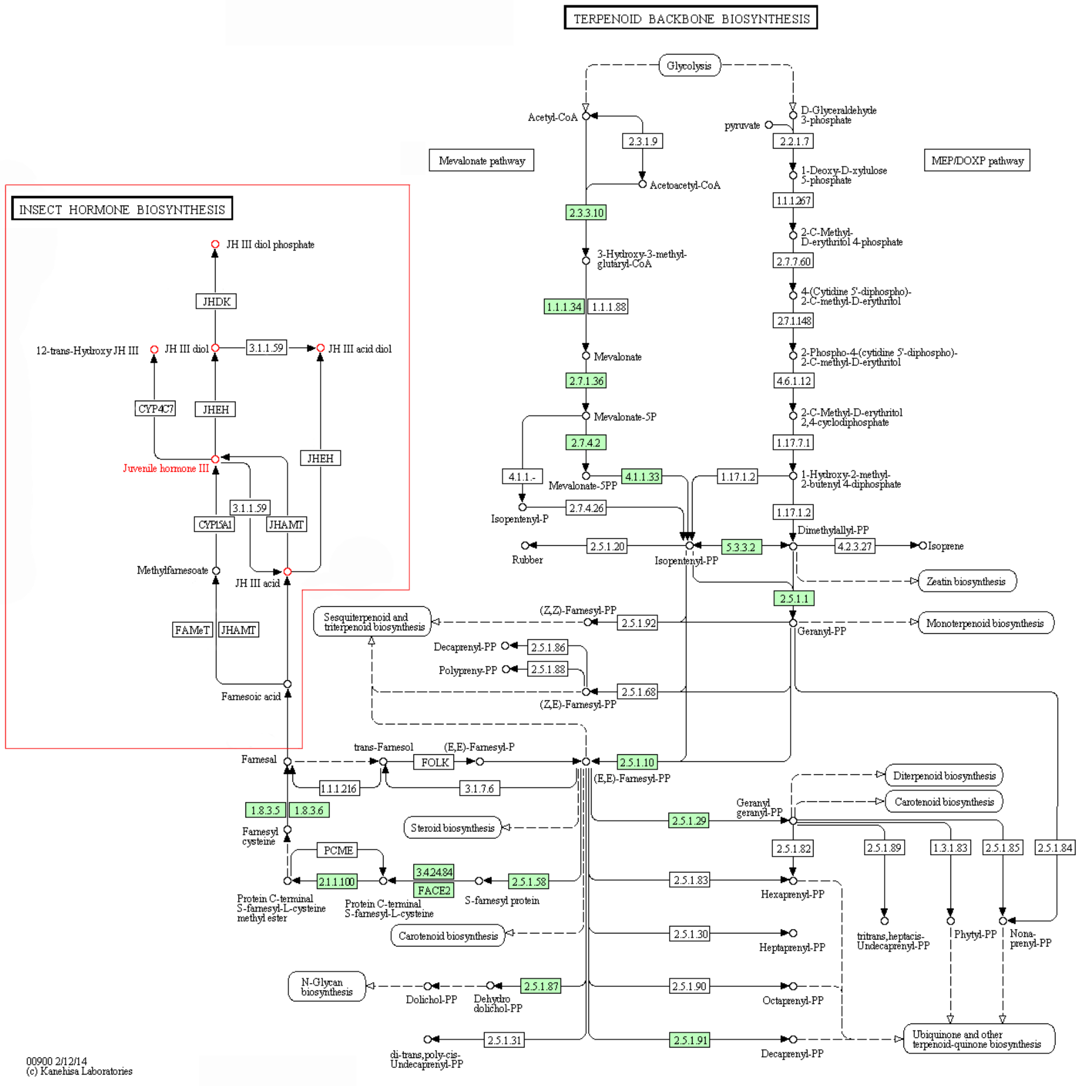

Supplementary Figure 9. The pathway in *Schistosoma japonicum* of *de novo* synthesis of JH from Acetyl-CoA. The pathway map is the combination of terpenoid backbone biosynthesis and insect hormone biosynthesis (red box) from KEGG pathway database. Genes in green box indicated the identical genes found in *de novo S. japonicum* transcriptome.

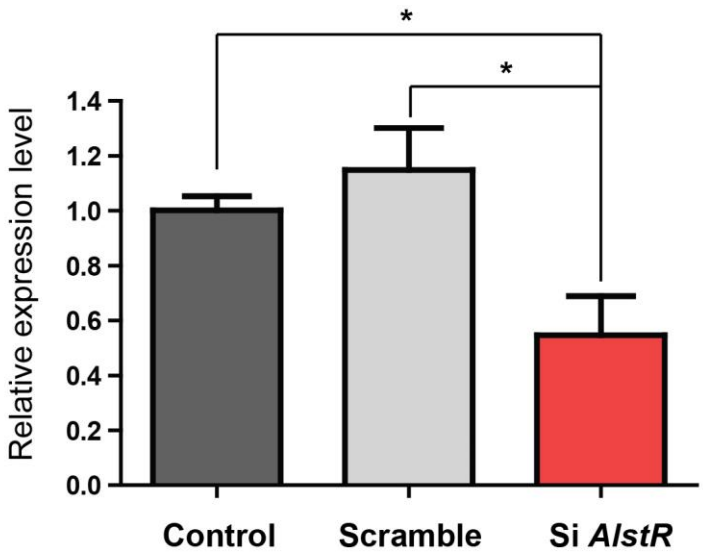

Supplementary Figure 10. The relative expression levels of *Sj AlstR* after *in vivo* RNAi. Worms were perfused from infected mice (20 dpi) that either injected with control plasmid, scramble plasmid or si AlstR expression plasmid for 72h. Females worms (5~8) from each mouse were separated from male and taken as a replicate. The expression level of the *Sj AlstR* was evaluated by RT-PCR with the *Sj PSMD* gene as a reference calibrator. Five independent experiments were performed and the expression level of *Sj AlstR* was reduced by ~50% in Si *AlstR* group compared to control or scramble group (mean±s.e.m., n=5). Student's t-test: * p<0.05.

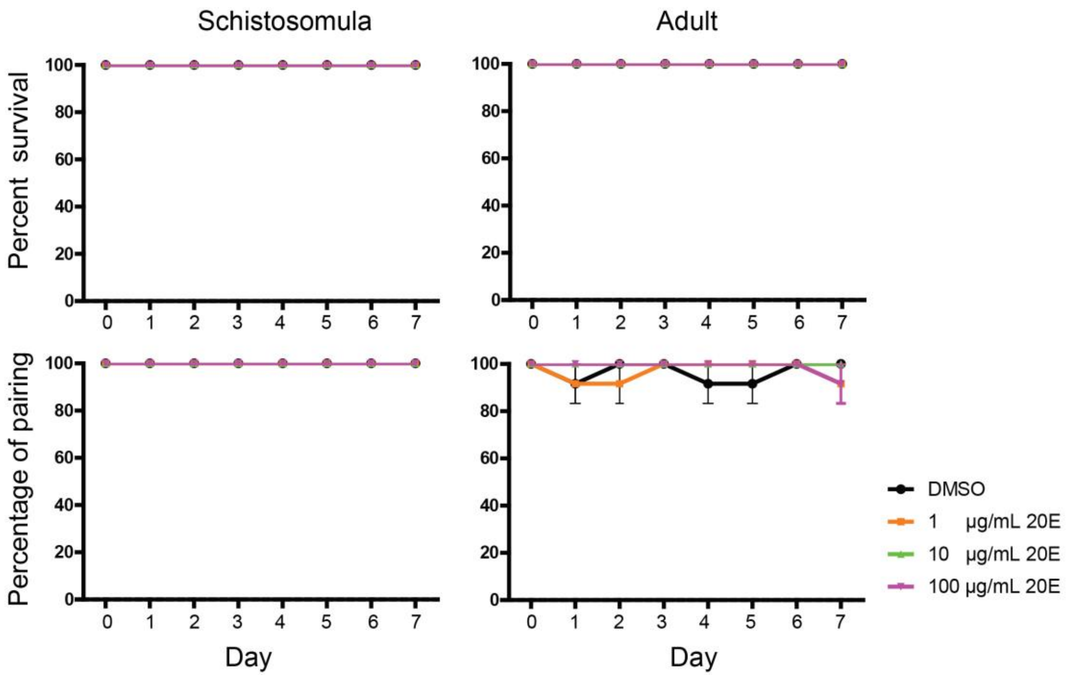

Supplementary Figure 11. The effect of 20E on the activities of *Schistosoma japonicum.* (mean±s.e.m., n=3)***.***

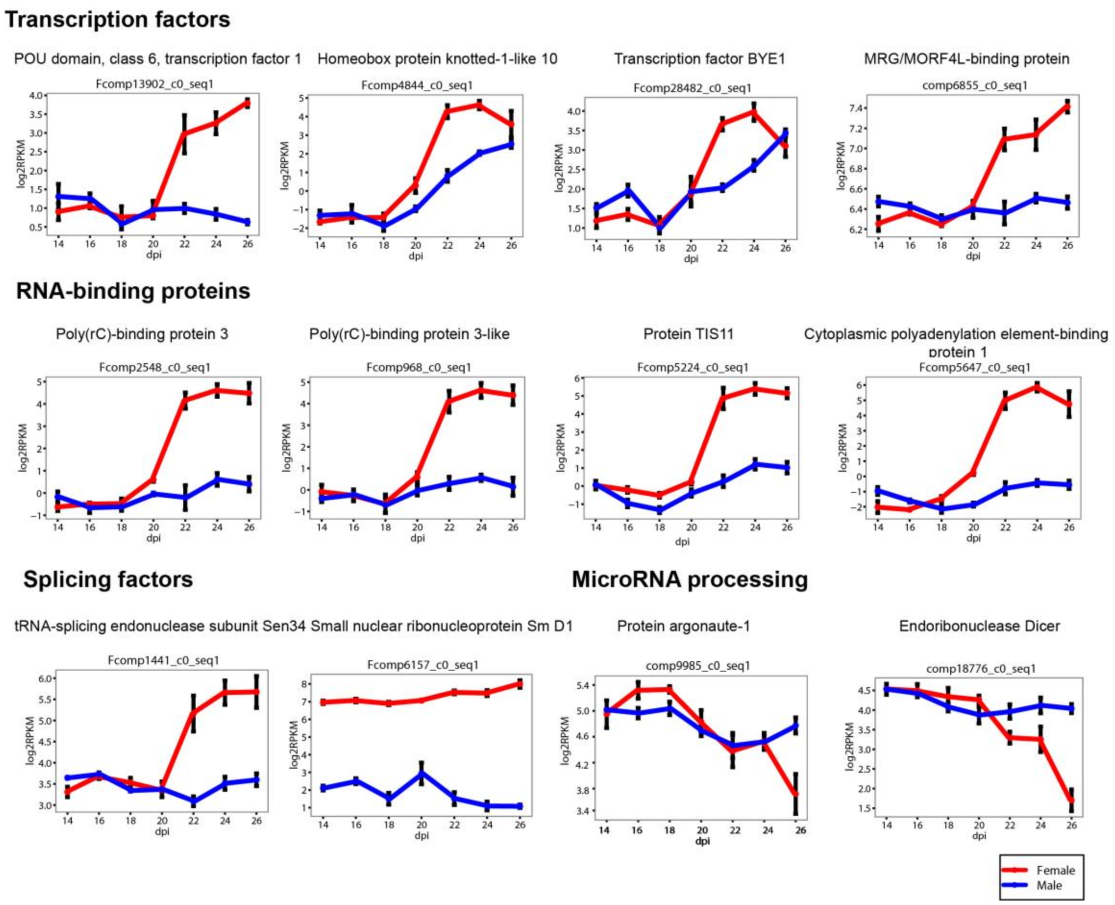

Supplementary Figure 12. The profiles of elements involved in regulation of gene expression. Y-axis represents the expression level (Log_2RPKM) of transcript in female (red) and male (blue). X-axis represents 7 different time points after infection. (mean± s.e.m., n=3).

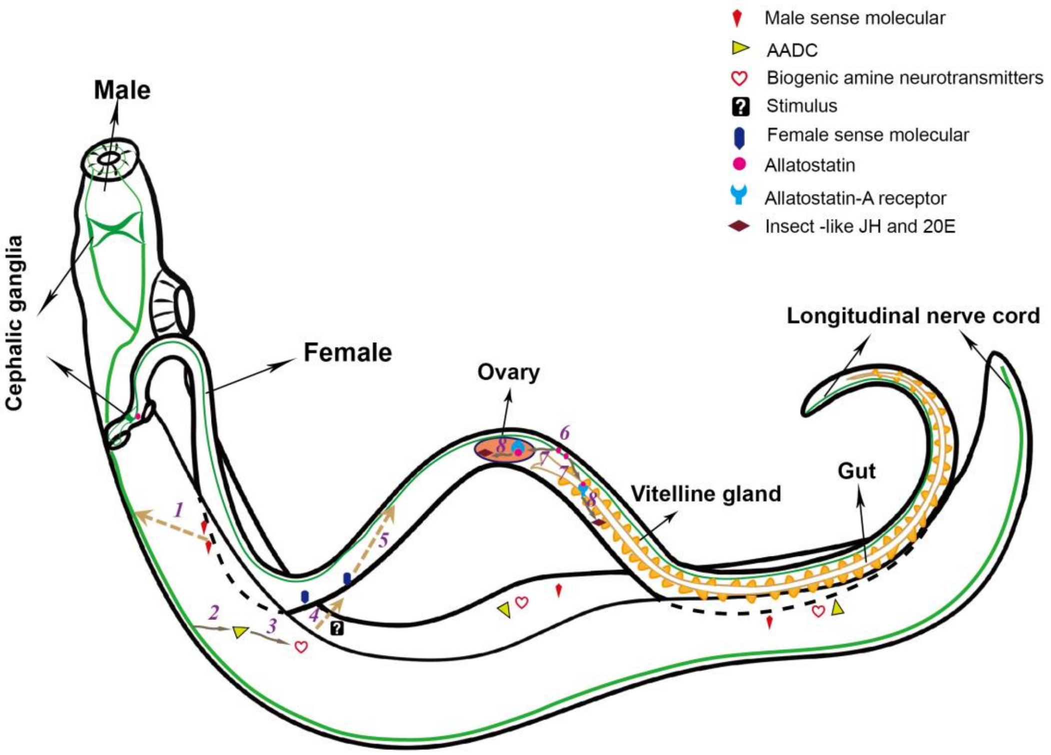

Supplementary Figure 13. Schematic overview of male-female interplay in schistosomes. 1. Stimulus indicating the presence of the female is transferred to the nervous system of the male schistosome by receptor molecules on the surface of the gynecophoral canal of the male. **2.** Biosynthesis of biogenic amines is enhanced by the over-expression of *SjAADC*. **3.** Biogenic amines regulate copulatory activity. **4.** The male transfers a tactile and/or chemical stimulus to the female during pairing. **5.** The female receives the stimulus through sensory molecules on the tegument. **6.** The female nervous system generates the neurotransmitter allatostatin. **7.** Allatostatin is transferred to the ovary and gut through the nervous system. **8.** Mediated by the allatostatin receptor, concentrations of insect-like hormones change, and this orchestrates the rapid development of the ovary and vitelline gland.

Supplementary Tables

Supplementary Table 1. The *S. japonicum* transcripts that matched to JH synthesis genes in insects.

Enzyme	Accession# insects	Accession# *S. japonicum*	Identity/e-value
**Farnesol Oxidase**	D2WKD9 *A. aegypti*	*AAW26955.1*	*29%,4e-17*
**Farnesal dehydrogenase**	*AGI96742* *A. aegypti*	*CAX73865.1*	*42%,1.2e-136*
**JH methyltransferase**		*NA*	*NA*
**JH epoxidase**		*NA*	*NA*

Supplementary Table 2. The cercarial infection doses at different time points.

Days post infection	14	16	18	20	22	24	26	28
**Cercarial infection doses**	800~ 1,000	600~ 800	500~ 600	200~ 250	150~ 200	100~ 150	80~ 100	80~ 100

Supplementary Table 3. Primer sequences for probe synthesis.

Gene name (Gene bank)	Primer name	Primer sequence
allatostatin A receptor like (AY814696.1)	Sense forward	5’TAATACGACTCACTATAGGGAGTCATTTAGTT3’
	Sense reverse	5’GACAAAATGGTAATGTAAATGTAGCGATTGTTTCA3’
	Antisense forward	5’AGTCATTTAGTTGGAAGTTTTCGTGCATATATTTCA3’
	Antisense reverse	5’TAATACGACTCACTATAGGGATGTAGCGA3’
aromatic-L-amino-acid decarboxylase (AY812557.1)	Sense forward	5’TAATACGACTCACTATAGGGGATTTCACTTATT3’
	Sense reverse	5’CATTTCAAAGTGACTTTTGTGTTGTTCAAATGC3’
	Antisense forward	5’GATTTCACTTATTGGGGCAAACAAATGATTG3’
	Antisense reverse	5’TAATACGACTCACTATAGGGGACTTTTGTGTT3’

Supplementary Table 4. The time point of each sample taken for RNA-seq

Sample#	Days post infection
Sj-male-1; Sj-male-2; Sj-male-3; Sj-female-1; Sj-female-2; Sj-female-3	14 dpi
Sj-male-4; Sj-male-5; Sj-male-6; Sj-female-4; Sj-female-5; Sj-female-6	16 dpi
Sj-male-7; Sj-male-8; Sj-male-9; Sj-female-7; Sj-female-8; Sj-female-9;	18 dpi
Sj-male-10; Sj-male-11; Sj-male-12; Sj-female-10; Sj-female-11; Sj-female-12	20 dpi
Sj-male-13; Sj-male-14; Sj-male-15; Sj-female-13; Sj-female-14; Sj-female-15	22 dpi
Sj-male-16; Sj-male-17; Sj-male-18; Sj-female-16; Sj-female-17; Sj-female-18	24 dpi
Sj-male-19; Sj-male-20; Sj-male-21; Sj-female-19; Sj-female-20; Sj-female-21	26 dpi
Sj-male-22; Sj-male-23; Sj-male-24; Sj-female-22; Sj-female-23; Sj-female-24	28 dpi
